# Synergistic Activity of Colistin Combined With Auranofin Against Colistin-Resistant Gram-Negative Bacteria

**DOI:** 10.3389/fmicb.2021.676414

**Published:** 2021-06-25

**Authors:** Xiaoxuan Feng, Shuai Liu, Yang Wang, Yulin Zhang, Lingxiao Sun, Haibo Li, Chunlei Wang, Yingmei Liu, Bin Cao

**Affiliations:** ^1^Graduate School of Peking Union Medical College, Chinese Academy of Medical Sciences, Peking Union Medical College, Beijing, China; ^2^Department of Pulmonary and Critical Care Medicine, Center for Respiratory Diseases, China-Japan Friendship Hospital, Beijing, China; ^3^China-Japan Friendship Hospital, National Clinical Research Center for Respiratory Diseases, Clinical Center for Pulmonary Infections, Capital Medical University, Beijing, China; ^4^Laboratory of Clinical Microbiology and Infectious Diseases, Department of Pulmonary and Critical Care Medicine, China-Japan Friendship Hospital, Beijing, China; ^5^Tsinghua University-Peking University Joint Center for Life Sciences, Tsinghua University, Beijing, China

**Keywords:** colistin, auranofin, colistin-resistant, repurposing, combination therapy, synergistic effect

## Abstract

Colistin-resistant (Col-R) bacteria are steadily increasing, and are extremely difficult to treat. New drugs or therapies are urgently needed to treat infections caused by these pathogens. Combination therapy with colistin and other old drugs, is an important way to restore the activity of colistin. This study aimed to investigate the activity of colistin in combination with the anti-rheumatic drug auranofin against Col-R Gram-negative bacteria. The results of checkerboard analysis demonstrated that auranofin synergized with colistin against Col-R Gram-negative bacteria. Time-kill assays showed significant synergistic antimicrobial activity of colistin combined with auranofin. Electron microscopy revealed that the combination resulted in more cellular structural alterations compared to each drug alone. Auranofin enhanced the therapeutic effectiveness of colistin in mouse peritoneal infection models. These results suggested that the combination of colistin and auranofin might be a potential alternative for the treatment of Col-R Gram-negative bacterial infections.

## Introduction

Polymyxins (including polymyxin B and colistin) are considered as last resort drugs for the treatment of infections caused by extensively drug-resistant Gram-negative bacteria. Polymyxins act on the outer membrane of Gram-negative bacteria, disrupt the stability of cell membrane, cause osmotic imbalance, and ultimately lead to cell death (Velkov et al., 2010; Berglund et al., 2015). Before 2015, mutations in chromosomal genes, such as *pmrAB, phoPQ*, and *mgrB*, were considered as the main causes of high-level polymyxin resistance (Olaitan et al., 2014). However, the discovery of plasmid-encoded *mcr-1* gene led to global reports of polymyxin resistance (Liu et al., 2016; Wang R. et al., 2018).

The gradual emergence of the polymyxin-resistant bacterial strains has greatly limited the antibiotic therapy options. It has been shown that repurposing FDA-approved non-antibiotic drugs in combination with polymyxin could be a promising alternative therapeutic strategy (Schneider et al., 2016; Ayerbe-Algaba et al., 2018, 2019; Cannatelli et al., 2018; Tran et al., 2018; Wang Y.M. et al., 2018; Zhou et al., 2018; Falagas et al., 2019; Hussein et al., 2020). To date, a number of FDA-approved non-antibiotic drugs, e.g., azidothymidine (Falagas et al., 2019), oxyclozanide (Ayerbe-Algaba et al., 2019), niclosamide (Ayerbe-Algaba et al., 2018), resveratrol (Cannatelli et al., 2018), mitotane (Tran et al., 2018), sertraline (Hussein et al., 2020), ivacaftor (Schneider et al., 2016), eugenol (Wang Y.M. et al., 2018), pterostilbene (Zhou et al., 2018), in combination with polymyxins have displayed synergistic killing against polymyxin-resistant Gram-negative bacteria. Auranofin is an FDA-approved anti-rheumatoid arthritis drug (Roder and Thomson, 2015). Adverse effects are associated with the long-term (months to years) use of auranofin, including diarrhea (40% of subjects), skin rashes (2–5%), hematologic abnormalities (rare), and proteinuria (5%; Kean et al., 1997). However, the most common side effect, diarrhea, can be easily managed (Suarez-Almazor et al., 2000). Hence, its largely acceptable toxicity paves the way to its repositioning for new and different therapeutic uses. Auranofin exerts a significant antimicrobial activity against numerous Gram-positive bacteria (Aguinagalde et al., 2015; Harbut et al., 2015; Tharmalingam et al., 2019), which is purported to inhibit the thioredoxin reductase (TrxR) of Gram-positive bacteria and disrupt the redox balance, resulting in cell death. However, auranofin alone has limited activity against Gram-negative bacteria (Harbut et al., 2015). Some studies have shown that colistin in combination with auranofin is effective against MDR Gram-negative bacteria (Sun et al., 2016; Torres et al., 2018). A recent study showed that auranofin could restore the activity of colistin against several *mcr-1* positive Enterobacteriaceae (Sun et al., 2020). However, it is not known if auranofin can restore the activity of colistin against Colistin-resistant (Col-R) Gram-negative bacteria of different species with different colistin resistance mechanisms. In this study, the *in vitro* activities of colistin in combination with auranofin were evaluated against a group of clinical Col-R Gram-negative bacteria including *Klebsiella pneumoniae, Escherichia coli, Pseudomonas aeruginosa*, and *Acinetobacter baumannii*. In addition, we explored the therapeutic effectiveness of colistin combined with auranofin in mouse peritoneal infection models.

## Materials and Methods

### Bacterial Isolates and Chemicals

We used 23 clinical Col-R isolates in this study. Four reference colistin-susceptible (Col-S) *K. pneumoniae* ATCC 700603, *E. coli* ATCC25922, *P. aeruginosa* ATCC27853, and *A. baumannii* ATCC19606 isolates were also used. Colistin and auranofin were purchased from the National Institutes for Food and Drug Control (Beijing, China). Colistin solutions were prepared in sterile Milli-Q water before the experiments. Stock solutions of auranofin were prepared in dimethyl sulfoxide (DMSO) and then diluted with sterile Milli-Q water to ensure a final DMSO concentration of ≤5% (v/v; Lin et al., 2018).

### *In vitro* Susceptibility Testing and Colistin Resistance Mechanisms

The minimum inhibitory concentrations (MICs) of colistin and auranofin were determined by the method described in the Clinical and Laboratory Standards Institute, 2019). Colistin or auranofin was prepared with two-fold serial dilutions. A final bacterial suspension at 5 × 10^5^ CFU/mL was added in each well, and incubated with two-fold serial dilutions of colistin or auranofin for 18 h at 37°C. MIC was determined as the lowest concentration that inhibited the visible growth of the bacteria. Colistin resistance mechanisms were analyzed with detection of *pmrA*, *pmrB*, *phoP*, *phoQ*, *mgrB*, *mcr-1*, and *mcr-8* genes by PCR (Liu et al., 2016; Wang X. et al., 2018; Ayerbe-Algaba et al., 2019). The sequences of the PCR products were determined by RuiBiotech (Beijing, China).

### Checkerboard Assays

The synergistic interaction between colistin and auranofin was tested using the checkerboard technique (Flamm et al., 2019). Colistin and auranofin were prepared with two-fold serial dilutions and mixed to create different concentration combinations. A final bacterial suspension at 5 × 10^5^ CFU/mL was added in each well. After incubation for 18 h at 37°C, the optimal fractional inhibitory concentration index (FICI) was calculated as the MIC of the combination divided by that of each compound used alone. FICI ≤ 0.5 denotes synergy, FICI > 0.5–4 denotes no interaction and FICI > 4 denotes antagonism (Odds, 2003).

### Time-Kill Assays

Time-kill assays were performed to further evaluate the synergistic effect of colistin with auranofin according to Hu’s method (Hu et al., 2019) with minor modifications. The concentration of colistin was used at 2 or 4 mg/L. The concentrations of auranofin were chosen according to the concentrations that showed synergistic effect with colistin in the checkerboard assays. Fresh cultures were mixed with colistin or auranofin alone or a combination and incubated at 37°C with shaking. Thereafter, 10 μL of each suspension was plated on nutrient agar plates for viable bacterial quantification after serial dilution at different time points of incubation. Synergistic activity was defined as a ≥2 log_10_ decrease in CFU/mL of the combination compared to the most active monotherapy (Doern, 2014).

### Scanning Electron and Transmission Electron Microscopy

The impact of colistin in combination with auranofin on the cellular morphology of the high-level Col-R *K. pneumoniae* 18605 and *A. baumannii* 13660 was examined by scanning electron microscopy (SEM) and transmission electron microscopy (TEM). For *K. pneumoniae* 18605, a log phase culture was treated with 2 mg/L colistin, 2 mg/L auranofin, or both for 2 h in cation-adjusted Mueller–Hinton broth (CAMHB). For *A. baumannii* 13660, a log phase culture was treated with 2 mg/L colistin, 4 mg/L auranofin, or both for 2 h in CAMHB. Bacterial pellets were obtained by centrifugation at 4,000 *g* for 10 min twice. Next, the pellets were fixed overnight at 4°C with 1 mL 2.5% glutaraldehyde. The fixatives were removed after centrifugation at 4,000 *g* for 10 min, and finally the bacterial pellets were resuspended in 1 mL PBS. SEM was performed with a HITACHI SU8020 scanning electron microscope. For TEM observation, all images were acquired using a JEM-1200EX microscope.

### *In vivo* Treatment Evaluation

To further evaluate the *in vivo* effect of colistin in combination with auranofin, two infection models were established in female ICR mice. 6–8 weeks old mice (weighing an average of 20 *g*) were purchased from Beijing Vital River Laboratory Animal Technology Co. Ltd. For the bacterial load experiment, A dose of 2 × 10^6^ CFU *K. pneumoniae* 18605 bacterial suspension was intraperitoneally (i.p.) injected into the mice. After 1 h of infection, mice were i.p. treated with vehicle, colistin sulfate (1 mg/kg) or auranofin (0.5 mg/kg) alone or their combination (*n* = 8 per group). All mice were euthanized at 14 h post-infection. Peritoneal fluid (PF) was collected by injecting 2 mL sterile saline solution into the peritoneum, followed by gentle massage and aspiration. The spleen was aseptically obtained, weighed and homogenized. Individual samples were serially diluted in sterile saline solution and 0.1 mL aliquots were placed on nutrient agar plates. The colonies were counted after incubation overnight at 37°C. The bacterial loads in PF and spleen between groups were compared using one-way ANOVA and the *post hoc* Bonferroni test. A *p*-value < 0.05 was considered significant.

For survival assay, mice were infected i.p. with 1 × 10^7^ CFU of *A. baumannii* 13660. After 0.5 h of infection, mice (*n* = 8 per group) were i.p. treated with vehicle or colistin sulfate (1.5 mg/kg) or auranofin (0.5 mg/kg) alone or in combination. The same treatment procedure was then repeated once daily until the end of the study. Survival rates were monitored for 5 days. Log-rank (Mantel–Cox) test was used to compared the survival distributions of different groups. A *p*-value < 0.05 was considered significant.

### Ethics Statement

The animal experiments were performed in accordance with the national standards for laboratory animals in China, with approval being provided by the Animal Ethical and Welfare Committee of the China-Japan Friendship Hospital (Zryhyy11-20-07-1).

## Results

### Synergistic Effect of Colistin and Auranofin in Checkerboard Assays

Minimum inhibitory concentrations of colistin and auranofin were tested against a group of 27 strains of four different Gram-negative bacterial species. As shown in Table 1, colistin MICs of the Col-R strains ranged from 4 to 1,024 mg/L, while those for the Col-S reference strains were 0.5–1 mg/L. MICs of auranofin were between 8 and 512 mg/L for all test strains. Mechanisms of resistance to colistin were identified in some of the strains studied (Table 1). Checkerboard assays revealed that the combination of colistin and auranofin showed a significant synergistic effect (FICI ≤ 0.5) against Col-R strains with different colistin resistance mechanisms. Additionally, the colistin MICs of most Col-R strains in the presence of auranofin were reduced to ≤2 mg/L. For all the Col-S reference strains, no interaction effect (FICI > 0.5) was observed.

**TABLE 1:** Checkerboard assays showing the effects of colistin combined with auranofin.

Pathogen	Strains	Source	Mechanism of colistin resistance	MIC(mg/L)				FICI
				Col	Aur	Col_Aur_	Aur_Col_	
*K. pneumoniae*	ATCC700603	ATCC	Colistin susceptible	1	512	0.5	8	0.516
	12959	BALF	PmrA G53V	64	128	0.5	4	0.039
	15979	Sputum	ISKpn14 at nt115 of *mgrB*	64	256	1	4	0.031
	13649	BALF	ISKpn14 at nt126 of *mgrB*	128	256	2	2	0.023
	13568	BALF	PmrA G53V	64	64	1	4	0.078
	18605	Wound	ISKpn14 at nt115 of *mgrB*	128	256	2	2	0.023
	18229	BALF	PhoQ A63E	256	512	1	4	0.012
	C505	Sputum	*mcr-8*	8	128	0.5	2	0.078
	C270	Urine	*mcr-8*	16	128	1	1	0.07
	17-R27	Liver	*mcr-1*	32	256	1	4	0.047
	09-20	Blood	*mcr-1*	16	256	0.5	2	0.039
*E. coli*	ATCC25922	ATCC	Colistin susceptible	0.5	512	0.5	2	1.004
	C297	Liver	*ND*	1024	32	0.5	8	0.25
	C1052	Urine	PmrB D283G	64	64	2	2	0.063
	C1157	Sputum	PhoQ V413F	64	64	1	1	0.031
	C1279	Urine	PmrA S29G	64	64	0.5	1	0.023
	C1461	Urine	PmrB Y358M, PhoP I44L	32	64	0.5	2	0.047
	17-R14	Liver	*mcr-1*	4	8	0.25	2	0.313
	08-85	Blood	*mcr-1*	8	16	1	1	0.188
*P. aeruginosa*	ATCC27853	ATCC	Colistin susceptible	1	512	1	2	1.004
	26751	BALF	PhoP V99L	128	512	4	4	0.039
	26587	BALF	PhoP V99L	512	512	2	8	0.02
	26683	BALF	ND	64	512	4	0.5	0.063
*A. baumannii*	ATCC19606	ATCC	Colistin susceptible	1	16	0.5	2	0.625
	13660	Sputum	PmrA T119S	512	32	2	4	0.129
	26701	Sputum	ND	512	128	1	4	0.033
	29831	Urine	ND	256	128	2	2	0.023

*BALF, bronchoalveolar lavage fluid; Col, colistin; Aur, auranofin; FICI, fractional inhibitory concentration index; and ND, not determined.*

### Time-Kill Assays of Colistin in Combination With Auranofin Against Col-R Strains

**FIGURE 1:**
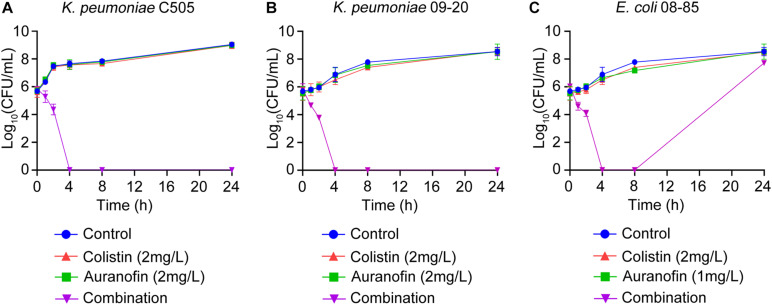
Time-kill assays showing the effects of colistin or auranofin alone or in combination against *mcr-8* positive *K. pneumoniae* C505 **(A)**, *mcr-1* positive *K. pneumoniae* 09-20 **(B)**, and *mcr-1* positive *E. coli* 08-85 **(C)**.

**FIGURE 2:**
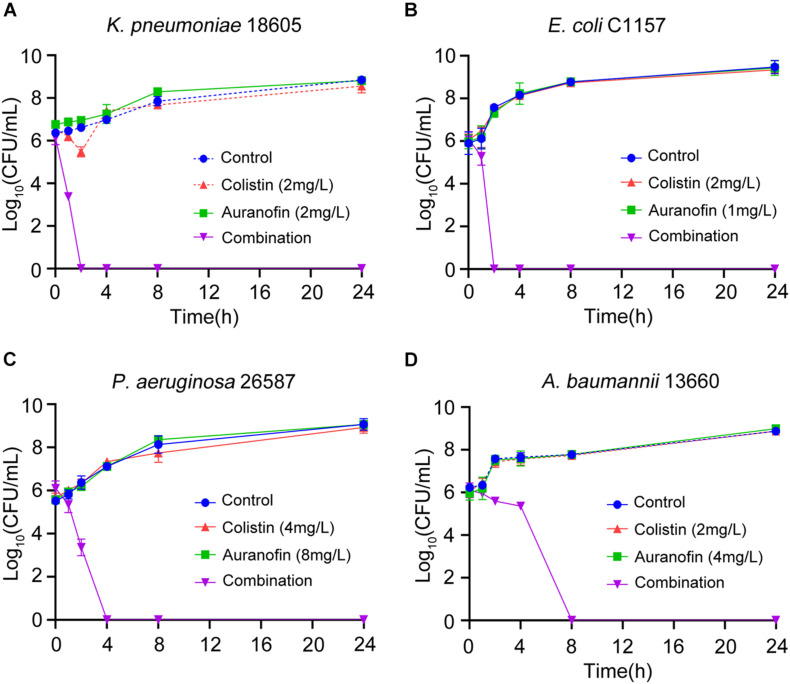
Time-kill assays showing the effects of colistin or auranofin alone or in combination against high-level Col-R *K. pneumoniae 18605* **(A)**, *E. coli* C1157 **(B)**, *P. aeruginosa* 26587 **(C)**, and *A. baumannii* 13660 **(D)**.

The synergistic effect of colistin in combination with auranofin was tested using time-kill assays against seven selected Col-R strains, namely *mcr-8* positive *K. pneumoniae* C505, *mcr-1* positive *K. pneumoniae* 09-20, *mcr-1* positive *E. coli* 08-85, high-level Col-R *K. pneumoniae* 18605, *E. coli* C1157, *P. aeruginosa* 26587, and *A. baumannii* 13660. As shown in Figure 1, for all three *mcr* gene positive isolates, neither colistin nor auranofin monotherapy showed any bacterial killing effect, and the growth curves were similar to that of control. The combination of colistin and auranofin resulted in the elimination of all three *mcr* gene positive isolates at 4 h, with no regrowth observed for *K. pneumoniae* C505 and *K. pneumoniae* 09-20. Interestingly, regrowth was observed for *E. coli* 08-85 after 8 h of treatment. As shown in Figure 2, for all the high-level Col-R isolates, auranofin monotherapy displayed no antimicrobial activity. Similar to auranofin, colistin monotherapy also could not prevent the growth of all high-level Col-R strains except *K. pneumoniae* 18605, wherein colistin monotherapy reduced the initial bacterial count slightly in the first 2 h, however, regrowth to the control value was observed at 24 h. When colistin was combined with auranofin, no viable bacterial cells were detected within 2–24 h for all high-level Col-R strains.

### Impact of Colistin and Auranofin on Cellular Morphology

**FIGURE 3:**
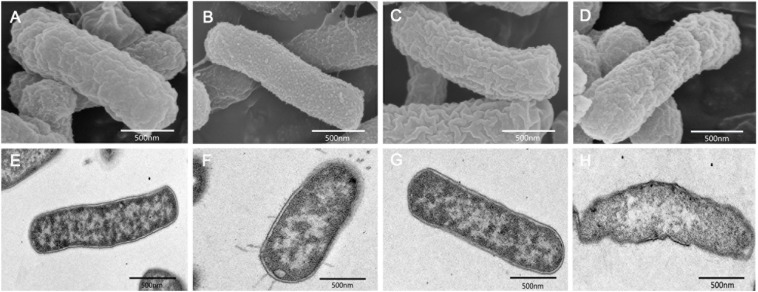
SEM and TEM images of high-level Col-R *K. pneumoniae* 18605 after treatment with 2 mg/L colistin alone **(B,F)**, 2 mg/L auranofin alone **(C,G)**, or combination **(D,H)** for 2 h. **(A,E)** represent the control condition.

We performed SEM and TEM experiments to determine the morphological changes of *K. pneumoniae* 18605 and *A. baumannii 13660* induced by colistin, auranofin, or both. For *K. pneumoniae* 18605, SEM images showed that auranofin monotherapy displayed no morphological changes (Figure 3C) compared to the control group (Figure 3A). Membrane blebbing was evidently observed with colistin monotherapy (Figure 3B). The combination treatment caused large-scale membrane disruptions (Figure 3D). By TEM, treatment with auranofin monotherapy did not show any impact on the cellular morphology (Figure 3G) compared to the control group (Figure 3E). However, minor protrusions were caused by colistin monotherapy (Figure 3F). With the combination, bacterial cell surface was extensively disrupted and showed cell lysis (Figure 3H). For *A. baumannii* 13660, SEM images showed that colistin (Supplementary Figure 1B) and auranofin (Supplementary Figure 1C) monotherapy displayed no morphological changes compared to the control group (Supplementary Figure 1A). The combination treatment (Supplementary Figure 1D) resulted in a significant reduction in cell length, and the cell surface was more uneven compared to the control group (Supplementary Figure 1A). For TEM, auranofin monotherapy (Supplementary Figure 1G) did not affected the cell surface. Membrane blebbing was observed with colistin monotherapy (Supplementary Figure 1F). Bacterial cells treated with the combination (Supplementary Figure 1H) were much shorter in length compared to the control group (Supplementary Figure 1E).

### Efficacy of Combination Therapy *in vivo*

**FIGURE 4:**
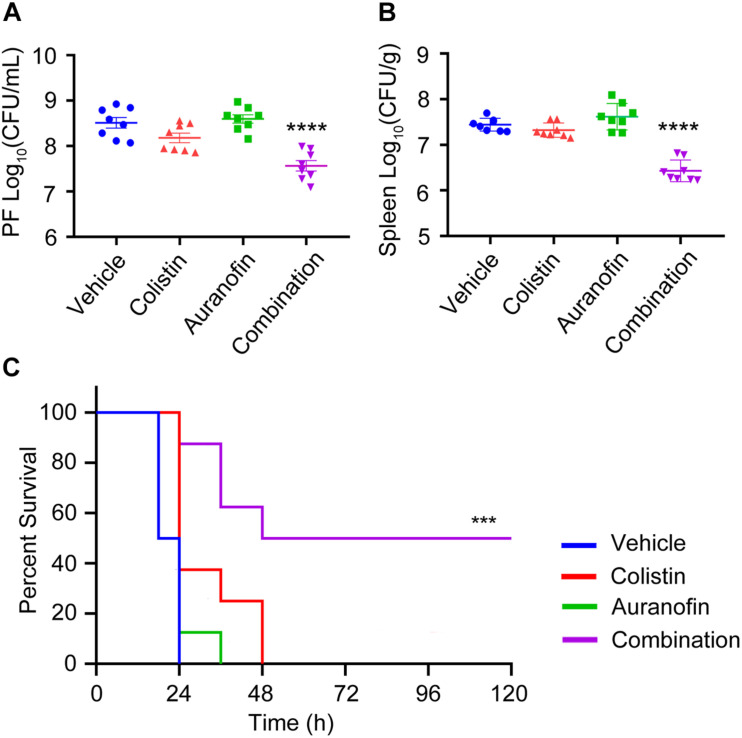
The combination of colistin and auranofin shows potency *in vivo*. **(A,B)** Mice were infected by high-level Col-R *K. pneumoniae* 18605 and received single dose of i.p. administration of vehicle, colistin sulfate, auranofin, or their combination (*n* = 8 per group). Bacterial loads in the PF **(A)** and spleen **(B)** are shown. **(C)** Survival curves showing combination efficacies in the peritoneal infection model. Mice were infected by a lethal dose of high-level Col-R *A. baumannii* 13660 and treated with one dose at 0.5 h post infection, followed by once-daily treatment with i.p. vehicle, colistin sulfate, auranofin, or the combination (*n* = 8 per group). ****indicates *p* ≤ 0.0001, ***indicates *p* ≤ 0.001, compared with the control group.

The *in vivo* efficacy of colistin in combination with auranofin was tested using two murine models of *K. pneumoniae* 18605 and *A. baumannii* 13660 infection. The dose of auranofin was selected based on the *in vitro* synergistic potentiation (Table 1 and Figures 1, 2), and was much lower than the maximum tolerated dose (Harbut et al., 2015). Colistin sulfate was dosed at approximately the human equivalent dose (Li et al., 2018). As shown in Figure 4, we evaluated the bacterial loads in the PF and spleen of mice. Neither colistin nor auranofin monotherapy showed antimicrobial activity against the infected bacteria (Figures 4A,B). In contrast, the combination of colistin and auranofin proved efficacious, with almost 10-fold bacterial load reduction (*p* < 0.0001) in the PF and spleen than that of the control group (Figures 4A,B). In the *A. baumannii* 13660 infection mouse model, monotherapy treatments administered 0.5 h post-infection did not demonstrate any significant survival beyond that of the control group (Figure 4C). However, animals receiving colistin and auranofin combination therapy daily for 5 days could rescue 50% of those treated (Figure 4C).

## Discussion

As a drug of last resort, colistin was used to treat infections caused by MDR Gram-negative bacteria. However, with the consumption of colistin, the number of Col-R strains increased (Bialvaei and Samadi Kafil, 2015). Since the discovery of *mcr-1* plasmid and its spread worldwide (Liu et al., 2016; Wang R. et al., 2018), the problem of colistin resistance has aroused widespread concern. Hence, it is important to restore the activity of colistin through combination therapy. In this study, we evaluated the potential application of colistin combined with auranofin to treat infections caused by Col-R isolates.

The *in vitro* results showed that auranofin exhibited significant synergy with colistin against Col-R Gram-negative bacteria including *mcr* positive strains. Similarly, a recent study reported that auranofin restored the activity of colistin against bacteria carrying *mcr-1* gene or its variants/homologs (Sun et al., 2020). Two previous studies reported that with the addition of auranofin, the effects of colistin, and auranofin against MDR Gram-negative bacteria were significantly increased (Sun et al., 2016; Torres et al., 2018).

*In vivo* study showed that colistin combined with auranofin improved the mice survival rate, and reduced the bacterial loads of *K. pneumoniae* 18605 in the PF and spleen of mice (Figures 4A,B). *In vitro* study on *K. pneumoniae* 18605 strain showed that the combined application of both colistin and auranofin at 2 mg/L dose could significantly reduce the initial bacterial count to the limit of detection (Figure 2A). However, the reduction of bacterial loads *in vivo* was much less than that in *in vitro* study. This discrepancy might be related to the pharmacokinetic properties of colistin and auranofin. The peak plasma concentration of colistin in mice treated with a single dose of colistin sulfate (2.5 mg/kg) was 3.08 mg/L (Li et al., 2018). Capparelli et al. (2017) have found that oral administration of 6 mg of auranofin daily for 7 days could result in a mean *C*_max_ value of 0.312 mg/L in plasma, which is far below its *in vitro* concentration of 2 mg/L used in this study. Using Monte–Carlo simulations, it has been shown that daily oral administration of 21 mg of auranofin could result in *C*_max_ of auranofin’s plasma concentration ranging between 0.4 and 1.6 mg/L (Capparelli et al., 2017). When mice were intraperitoneally administered with a single dose of 1 mg/kg colistin sulfate and 0.5 mg/kg auranofin, the plasma concentration of each compound might not reach the corresponding *in vitro* concentration. Therefore, the combination therapy showed less antimicrobial activity *in vivo* than *in vitro*. The safety of auranofin in clinical use is well established. Auranofin has been extensively used in clinical setting at the FDA-approved human dose (6 mg/day), a steady-state blood gold concentration of 3.5 μM would be reached in 12 weeks (Debnath et al., 2012). Patients with rheumatoid arthritis who were treated with auranofin (6 mg/day), had shown a safe toxicity profile (Suarez-Almazor et al., 2000). Patients with relapsed chronic lymphocytic leukemia who received 9 and 12 mg of auranofin daily for at least 28 days, followed by up to 21 mg/day, were well-tolerated (Clinical Trails registration no. NCT01419691). Given that the course of antimicrobial therapy is much shorter than that of treatment for rheumatoid arthritis or chronic lymphocytic leukemia, auranofin could be safely used to treat bacterial infections.

Scanning electron microscopy and TEM of *K. pneumoniae* 18605 and *A. baumannii* 13660 cells revealed that auranofin alone had no influence on cellular morphology (Figures 3C,G and Supplementary Figures 1C,G). Membrane blebbing was evidently observed when the cells were treated with colistin alone (Figure 3B and Supplementary Figure 1F). Similar changes have been observed in *E. coli* (Koike et al., 1969), *P. aeruginosa* (Hussein et al., 2020) after treatment with colistin alone. The bacterial outer membrane of *K. pneumoniae* 18605 was dramatically disrupted by the combination of colistin with auranofin, and showed cell lysis (Figures 3D,H). For *A. baumannii* 13660, the combination affected the overall structure of the strain, leading to an extensive shortening in the length of the bacteria (Supplementary Figures 1D,H). These observations suggested that auranofin may directly enhance the structural alteration effects of colistin.

Auranofin directly inhibited the TrxR in *S. aureus* and *M. tuberculosis*, leading to disruption of thiol-redox homeostasis and cell death (Harbut et al., 2015). However, the presence of glutathione system in several Gram-negative bacteria could maintain the redox balance and render auranofin ineffective (Harbut et al., 2015). A previous study has reported that the permeability barrier of outer membrane is the reason for the lack of activity of auranofin against Gram-negative bacteria (Thangamani et al., 2016). For the *mcr-1* positive bacteria, auranofin was reported that irreversibly inhibited the function of MCR-1 via displacement of Zn (II) cofactors from the active site, thereby resensitizing *mcr-1* positive bacteria to colistin (Sun et al., 2020). MCR-1 is a phosphoethanolamine (PEA) transferase, which confers colistin resistance by adding the PEA moiety to lipid A (Wang Y. et al., 2017). Further research is needed to identify the exact mode of action of auranofin when combined with colistin against high-lever Col-R pathogens.

In summary, auranofin enhanced the *in vitro* and *in vivo* antimicrobial activities of colistin against Col-R Gram-negative bacteria. As auranofin is currently available, it can be easily used for antimicrobial therapy instead of developing new antimicrobial drugs. There is great potential for this novel combination to treat infections caused by Col-R Gram-negative bacteria.

## Data Availability Statement

The original contributions presented in the study are included in the article/Supplementary Material; further inquiries can be directed to the corresponding author/s.

## Ethics Statement

The animal study was reviewed and approved by Animal Ethical and Welfare Committee of the China-Japan Friendship Hospital (Zryhyy11-20-07-1).

## Author Contributions

BC and YL conceived and designed the study. XF, SL, YW, YZ, LS, HL, and CW carried out the experiments. XF performed the statistical analysis and wrote the draft. All authors read and approved the final version of the manuscript.

## Conflict of Interest

The authors declare that the research was conducted in the absence of any commercial or financial relationships that could be construed as a potential conflict of interest.
