# Supplementary material for: Synergistic Activity of Colistin Combined With Auranofin Against Colistin-Resistant Gram-Negative Bacteria
Source: Front Microbiol. 2021 Jun 25;12:676414. doi: 10.3389/fmicb.2021.676414 (PMC8267823; doi:10.3389/fmicb.2021.676414)
Supplement: Supplementary file 1 [file Image_1.pdf]

Supplemental materials

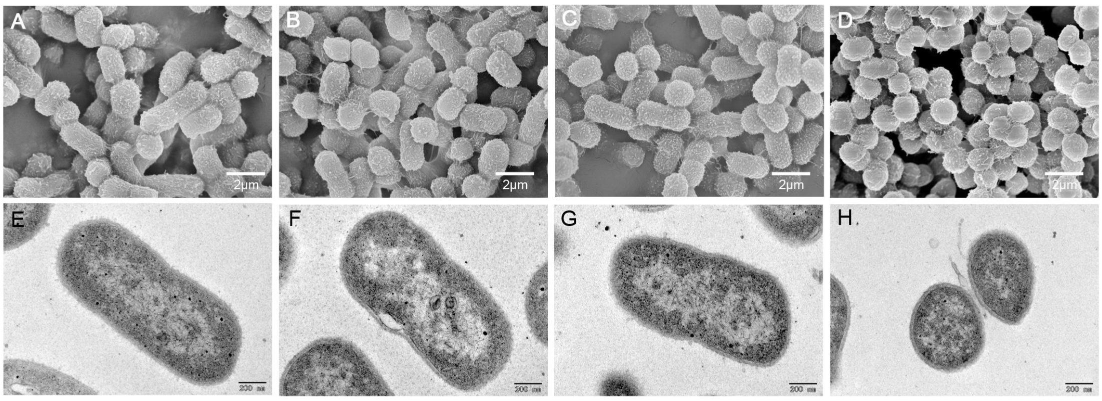

Supplemental Figure 1 SEM and TEM images of high-level Col-R *A. baumannii 13660* after treatment with 2 mg/L colistin alone (B, F), 4 mg/L auranofin alone (C, G) or combination (D, H) for 2 h. A and E represent the control condition.
